# Epigenome Microarray Platform for Proteome-Wide Dissection of Chromatin-Signaling Networks

**DOI:** 10.1371/journal.pone.0006789

**Published:** 2009-08-26

**Authors:** Dennis J. Bua, Alex J. Kuo, Peggie Cheung, Chih Long Liu, Valentina Migliori, Alexsandra Espejo, Fabio Casadio, Christian Bassi, Bruno Amati, Mark T. Bedford, Ernesto Guccione, Or Gozani

**Affiliations:** 1 Department of Biology, Stanford University, Stanford, California, United States of America; 2 Institute of Molecular and Cell Biology, Singapore, Singapore; 3 University of Texas M.D. Anderson Cancer Center, Smithville, Texas, United States of America; 4 Department of Experimental Oncology, European Institute of Oncology, Milan, Italy; University of Munich and Center of Integrated Protein Science, Germany

## Abstract

Knowledge of protein domains that function as the biological effectors for diverse post-translational modifications of histones is critical for understanding how nuclear and epigenetic programs are established. Indeed, mutations of chromatin effector domains found within several proteins are associated with multiple human pathologies, including cancer and immunodeficiency syndromes. To date, relatively few effector domains have been identified in comparison to the number of modifications present on histone and non-histone proteins. Here we describe the generation and application of human modified peptide microarrays as a platform for high-throughput discovery of chromatin effectors and for epitope-specificity analysis of antibodies commonly utilized in chromatin research. Screening with a library containing a majority of the Royal Family domains present in the human proteome led to the discovery of TDRD7, JMJ2C, and MPP8 as three new modified histone-binding proteins. Thus, we propose that peptide microarray methodologies are a powerful new tool for elucidating molecular interactions at chromatin.

## Introduction

Chromatin structural dynamics regulate diverse cellular functions that influence survival, growth, and proliferation. Disruption of chromatin homeostasis is thought to fundamentally impact on the development and progression of cancers and other diseases. One of the major mechanisms for regulating chromatin structure involves the reversible covalent post-translational modification (PTM) of histone proteins by chemical moieties such as acetyl-, methyl- and phospho- groups. These chemical marks are proposed to constitute an epigenetic code that can be maintained in dividing cells and inherited across generations. Combinations of different histone modifications are linked to discrete chromatin states and are thought to regulate the accessibility of DNA to transacting factors [1], [2]. At the molecular level, histone marks can act as ligands for modular protein domains found on chromatin-regulatory proteins [3], [4]. In this context, the proteins and domains that recognize histone modifications, named “effectors” or “readers”, are thought to define the functional consequences of many classes of modifications by transducing molecular events at chromatin into biological outcomes.

Critical insight into how domain recognition for histone modifications influences chromatin activities has come from the identification and characterization of methyl-lysine effectors. Because methylation does not neutralize the charge of the modified residue nor does addition of methyl groups add considerable bulk, this mark is believed to create a distinct molecular architecture on histones that is then recognized by specialized binding domains (e.g. chromodomains (CD) and Plant Homeodomain (PHD) fingers) present within chromatin-regulatory proteins. For example, components of repressive complexes, such as heterochromatin protein 1 (HP1), contain CDs that allows them to specifically recognize the appropriate repressive methylation mark, histone H3 trimethylated at lysine 9 (H3K9me3). Similarly, histone H3 trimethylated at lysine 4 (H3K4me3), which is postulated to enhance transcriptional activation due to its enrichment near the transcriptional start site of active genes [5]–[7], is recognized by several modules found on factors associated with transcriptional activation [8], [9]. However, H3K4me3 is also a ligand for complexes with very different activities, such as transcriptional repression [10] and recombination [11], [12]. Taken together, the biological outcomes of histone marks are impacted by both their location in chromatin regions and the repertoire of effectors that have access to those regions. While several effector modules have been discovered for H3K4me3 and H3K9me3, many other marks have few or no known effectors. Since characterization of effector domain interactions with histone state-specific ligands has been instrumental in unraveling chromatin-signaling networks, it is important to develop new methods that allow for a systematic, high-throughput way to identify novel histone mark sensors.

Here we describe the development, validation, and application of a human epigenome peptide microarray platform (HEMP) for high-throughput identification of ligands for effector modules. We have probed this platform with modification-specific antibodies and known chromatin effector domains to test the integrity of the individual peptide features on the slides. Furthermore, we screened a large library of Royal Domain family members and identified three modules (the chromodomain of MPP8 (MPP8_CD_) and the tudor domains (TD) of TDRD7 (TDRD7_TD_), and JMJ2C (JMJ2C_TD_)) with novel modified-histone binding activity. Taken together, our results demonstrate that the technology platform described here can, broadly, contribute to the unraveling of epigenetic mechanisms and, more specifically, facilitate molecular dissection of chromatin signaling networks.

## Results

### Human epigenome peptide array construction and validation

**Figure 1:**
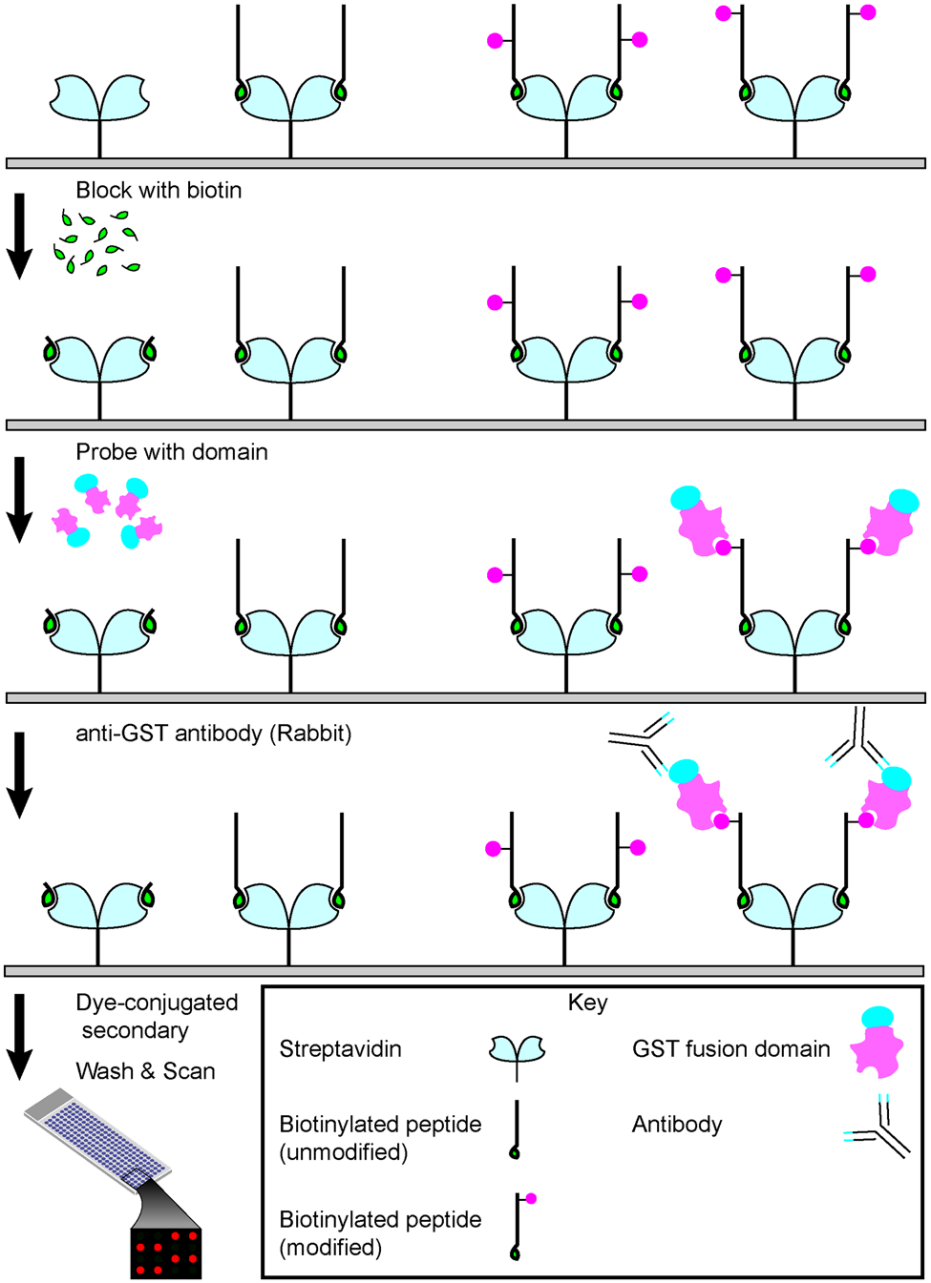
Key steps in the human epigenome peptide microarray (HEMP) procedure.

To generate HEMP as a tool for characterization and discovery of chromatin effectors, we first synthesized a large collection of biotinylated histone peptides of approximately 20 amino acids in length. The peptides correspond to regions of human histone proteins that are either unmodified or contain a single modification (acetyl-, methyl-, or phosphoryl- moieties) at known PTM sites (Table S1). The quality of all the peptides used in the study was confirmed by mass spectrometry and dot-blot analyses (data not shown). Notably, the majority of lysine residues known to be methylated or acetylated on histones in humans are represented in this library, including all methyl-lysine states detected to date on histone H3. The modified peptide features were spotted onto streptavidin-coated slides, incubated with an antibody or effector domain of interest, and then the antibody or effector domain was visualized as schematized (Fig. 1). Peptides were secured to slides by biotin-streptavidin interactions rather than other types of slide surfaces to direct the orientation of peptides and to provide sufficient space from the surface to allow for ligand-recognition (data not shown).

**Figure 2:**
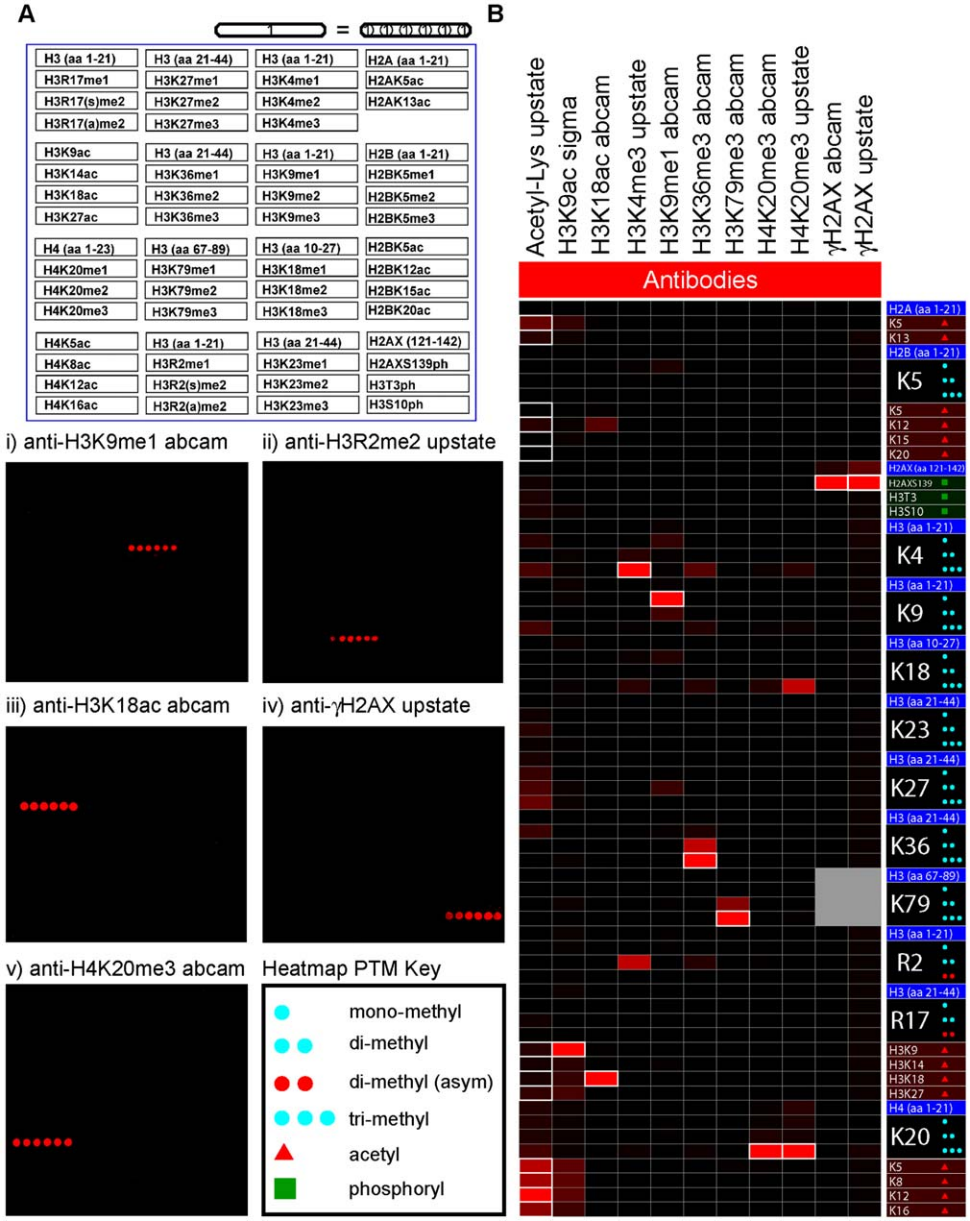
HEMP slides as a diagnostic tool for testing antibody specificity. (a) Array images for antibodies: i) anti-H3K9me1, ii) anti-H3R2me2 (asymmetric), iii) anti-H3K18ac, iv) anti-γH2AX, and v) anti-H3K20me3 with schematic of array layout and key. (b) Heatmap representation of antibody HEMP slide data (See Table S2 for additional antibody details). The epitope(s) that the antibody was generated against is/are highlighted with a white border. See “Heatmap PTM key” for details about peptides with post-translational modifications (PTMs). Note, that di-methyl arginine residues with blue circles are symmetrically di-methylated. SNR RN, signal-to-noise ratio range-normalized. n.t., not tested.

First HEMP arrays were probed with a number of commercially available antibodies commonly used in the literature to validate the integrity of the spotted peptides (Table S2). We chose antibodies that represent the different classes of modifications present on the array (lysine and arginine methylation, phosphorylation, and acetylation). As shown in Figure 2a, the peptide detected upon array probing was consistent with the epitope specificity designated in the product data sheets provided with the various antibodies. For example a γH2AX antibody bound specifically to H2AX peptides (residues 121–142) phosphorylated on Ser139, but the antibody did not significantly recognize the unmodified H2AX peptide or the other sixty peptides spotted on the array (Fig. 2a(iv)). Similarly, antibodies raised against monomethylated H3K9 (Fig. 2a(i)), asymmetric dimethylated H3R2 (Fig. 2a(ii)), trimethylated H4K20 (Fig. 2a(v)), and acetylated H3K18 (Fig. 2a(iii)) detected most strongly the appropriate peptides without appreciably cross-reacting with other peptides present on the HEMP slide. Based on these data, we conclude that the printing of the peptides onto slides as such does not disrupt peptide integrity since several independent epitopes are adequately recognized by a number of antibodies.

To facilitate a quantitative interpretation of array data, for instance for factors (antibodies and protein modules) that recognize more than one epitope, we utilized heatmap representations (Fig. 2b), which have been widely used for the presentation of nucleotide array data. Peptide array data was converted into heatmaps by determining the signal-to-noise ratio (SNR) at all features, with the values range-normalized (range 0–100) to address intrinsic variations in antibody affinities (see methods). As an example, an acetyl-lysine antibody was probed on our platform to determine the relative affinity of this antibody for the fourteen acetylated histone peptides present on the arrays. As shown in Figure 2b, this antibody, while detecting a broad spectrum of acetylated residues, preferentially recognizes acetyl-lysines present on histone H4 in comparison to acetyl-lysines found on H2A, H2B, or H3. Analyzing peptide array data in heatmap-form additionally allows for quick assessment of antibody cross-reactivity. For example, an antibody raised against a H3K79me3 antigen that is documented to recognize both H3K79me3 and H3K79me2, detects both H3K79me3 and H3K79me2 peptides when tested on HEMP slides (Fig. 2b). Inspection of the heatmap, however, indicates that the two epitopes are not recognized equally; a stronger signal is observed with H3K79me3 peptides relative to H3K79me2, and H3K79me1 is not detected (Fig. 2b). Appropriate interpretation of many techniques in chromatin biology – such as chromatin immunoprecipitation (ChIP) assays, relies on the availability of highly specific antibodies. Our data demonstrate the utility of using HEMP arrays as a tool to obtain a comprehensive, unbiased assessment of the relative specificities of newly developed antibodies prior to their use in downstream applications.

### Detection of modification-dependent binding activity by known methyl-lysine effector modules

**Figure 3:**
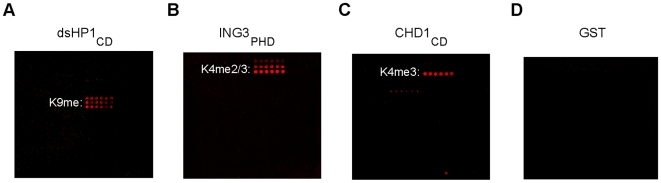
Detection of known chromatin effector-histone PTM interactions using HEMP slides. (a) The chromodomain of *Drosophila melanogaster* heterochromatin protein 1 alpha (dsHP1_CD_), (b) the plant homeodomain of human inhibitor of growth 3 (ING3_PHD_), and (c) the double chromodomains of human chromodomain-helicase-DNA-binding protein 1 (CHD1_CD_), all recognize, as indicated, their cognate histone ligand on the peptide array. All these protein domains are expressed as GST-fusions and an array probed with GST alone (d) serves as a negative control. For order of peptide spotting, see schematic in Figure 2a.

Next, we probed slides with protein modules that have known methyl-lysine binding activity. As shown in Figure 3a, the CD of *Drosophila melanogaster* heterochromatin protein 1 (HP1) alpha, the PHD finger of human inhibitor of growth 3 (ING3), and the double CDs of human Chromodomain-helicase-DNA-binding protein 1 (CHD1) all bind to their cognate histone ligand. Although this assay cannot be used to measure kinetic parameters, a comparison of interaction intensities detected on peptide arrays with published dissociation constants between several effectors and various histone ligands, a general pattern emerges suggesting that interactions that are ≤150–300 uM Kd range can be detected on the arrays (Fig. 3; Table S3; data not shown). Even though weak interactions will not be discerned (Kd > 300 uM), we conclude that this technology is suitable for proteomic-scale identification of most physiologically relevant protein PTM sensors, since virtually all published effector-PTM interactions are within the detection limit of HEMP technology.

### High-throughput identification of novel methyl-lysine effector modules

**Figure 4:**
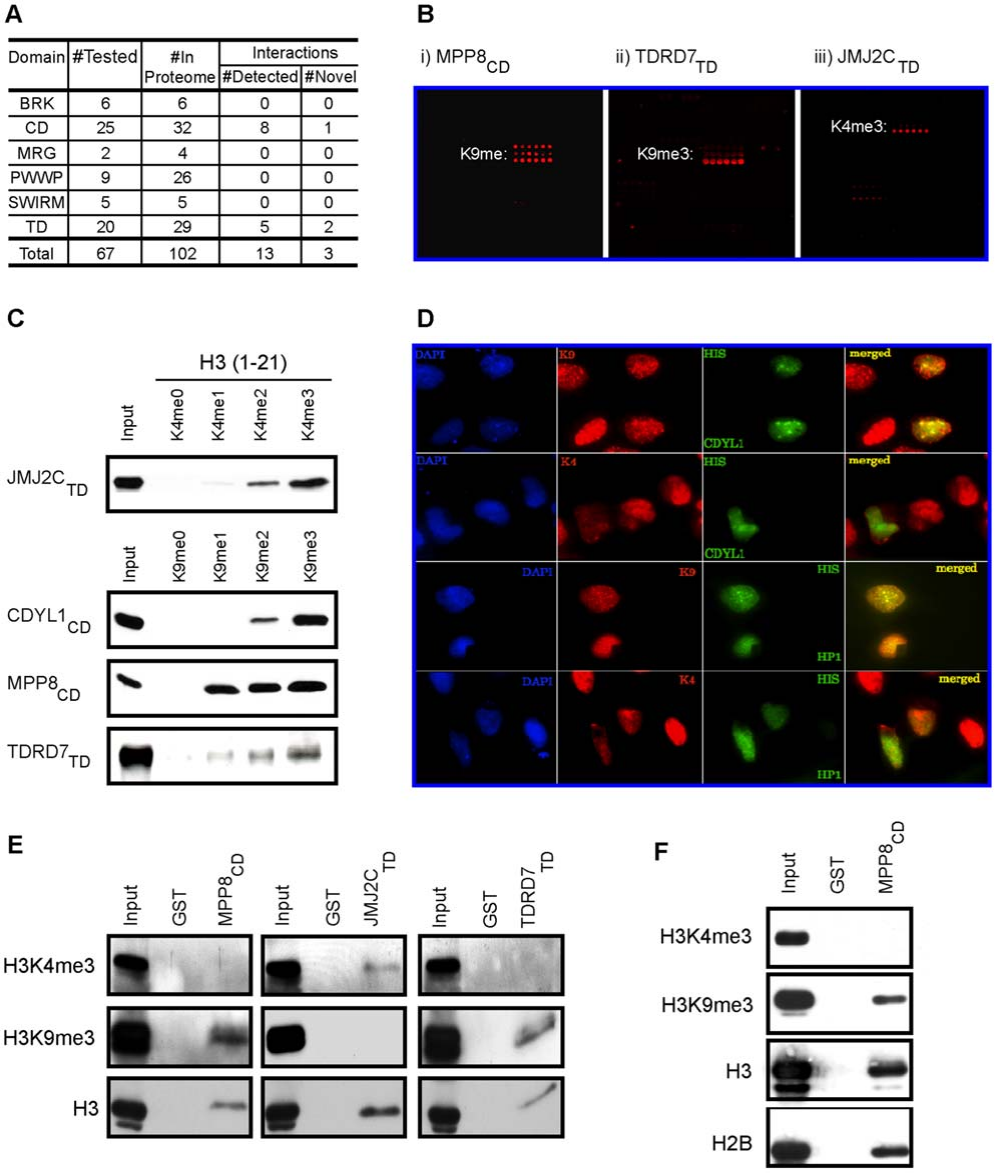
Identification of three novel methyl-histone binding modules. (a) Table summarizing the number of domains tested in this study and the number of interactions detected. CD, chromodomain. TD, tudor domain. (b) Array images for: i) MPP8_CD_, ii) TDRD7_TD_, and iii) JMJ2C_TD_. Peptide/s detected in each experiment is indicated. See Figure 2a for array schematic. (c) Validation of array results in peptide-binding assays. Biotinylated peptide pull-down assay using peptides detected in (b) and the indicated GST-fusion proteins. (d) Co-localization of CDYL1 with H3K9me3. Representative immunofluorescence images of U2OS cells transfected with His-tagged CDYL1 and co-stained with the indicated antibodies. K9  =  H3K9me3, K4  =  H3K4me3. (e) Validation of array results in bulk-histone binding assays. Calf-thymus histone pull-down with the indicated proteins: MPP8_CD_, TDRD7_TD_, and JMJ2C_TD_. In each case the domain was pulled-down and the pellet was probed with the indicated antibodies. (f) MPP8_CD_ binds to HeLa-purified nucleosomes enriched for H3K9me3 but not H3K4me3. Pull-downs of GST or GST-MPP8_CD_ protein after incubation with HeLa nucleosomes were probed with the antibodies indicated (see Fig. S1 for quantitation).

In previous work we demonstrated the ability of pilot versions of the peptide microarray platform to identify novel ligands for a number of specific protein modules [11], [13]–[15]. To test the contemporary HEMP slides in a proteome-wide discovery context, we generated an expression library of chromatin-associated domains consisting primarily of Royal super-family members (Table S4). The Royal super-family, defined by conservation of sequence and structural elements, includes CDs, TDs, MBT (malignant brain tumor) repeats, plant Agenet, and PWWP domains [16]. Within this family, there are a number of established methyl-lysine and methyl-arginine effector domains. Further, it is likely that amongst the numerous uncharacterized members of the family, new effectors await discovery. Therefore, we reasoned that the Royal family – consisting of known and candidate effectors modules, is a promising group of domains to screen on the arrays. In addition, the majority of BRK, MRG, and SWIRM domains found in the human proteome were included in our screen because these motifs are commonly co-present with Royal family domain-containing chromatin-associated proteins. We used two additional criteria for testing specific domains included in the expression library: (i) domains with known binding activity to serve as positive controls and (ii) domains found on proteins that are implicated in human disease. To this end, we tested approximately seventy distinct domains, including the majority of the Royal family members present in the human proteome (Fig. 4). As summarized in Figure 4a, a large majority of chromodomains (25/32) was screened, and out of these, eight positives were detected – seven of which had previously been described [17]–[21], and one novel interaction for the protein MPP8 was discovered (Fig. 4b(i); Table 1; see below). We failed to detect association of the MRG15_CD_ with H3K36me [22], though we note that there are alternatively spliced forms of this domain that might have different activity. Similarly, we tested a version of CDYL1_CD_ that bound to H3K9me3 *in vitro*, in contrast to a differentially spliced version that was reported to bind weakly [23] (Fig. 4c). Next we tested for co-localization of the full-length CDYL1 protein (harboring the version of the CD tested in our library) with histone marks *in vivo* (Fig. 4d). These localization experiments support our *in vitro* data since CDYL1 co-localizes with H3K9me3, but not H3K4me3. Taken together, 25% of chromodomains in the human proteome have clearly detectable histone methyl-lysine binding activity. The remaining majority (75%) of human chromodomains might only recognize histone PTMs in the context of nucleosomes and thus would not be detected on peptide arrays. Or these CDs may have altogether different activities such as recognition of a methylated non-histone protein or a different non-histone molecular ligand like RNA [24] (see discussion).

**Table 1:** Modification-dependent interactions detected using HEMP arrays.

Protein	Domain	H3K4me	H3K9me	H3K27me	H4K20me	Reference
CHD1	CD	+	−	−	−	[21], [38]
CDY1	CD	−	+	+	−	[23], [26]
CDYL1	CD		+	+	−	[23], [39]
CDYL2	CD	−	+	+	−	[23]
HP1α	CD	−	+	−	−	[17], [18]
HP1β	CD	−	+	−	−	[26]
HP1γ	CD	−	+	−	−	[26]
MPP8	CD	−	+	−	−	This study
53BP1	TD	−	−	−	+	[25], [26]
JMJ2A	TD	+	−	−	+	[26], [27]
JMJ2C	TD	+	−	−	−	This study
PHF20	TD	−	−	−	+	[26]
TDRD7	TD	−	+	−	−	This study

CD  =  chromodomain; TD  =  tudor domain.

Twenty of twenty-nine tudor domains present in the human proteome were also investigated (Fig. 4a; Table 1). Within this group were three known methyl-lysine-binding TDs (53BP1, PHF20, and JMJ2A [25]–[27]) as well as a TD with binding specificity for non-histone arginine methylated substrates (SMN [26], [28]). Accordingly, 53BP1, PHF20, and JMJ2A bound to their known ligands, while SMN did not bind any of the methyl-histone peptides – including arginine methylated peptides (Table 1). Of the sixteen uncharacterized tudor domains, we observed that two new tudor domains (TDRD7 and JMJ2C) have methyl-histone binding activity (Fig 4b; see below). We also tested several additional domains, including MRG, BRK, SWIRM, and PWWP domains, but no binding was detected. Thus, 25% of the tudor domains in our library have histone methyl-lysine binding activity, and several other domains did not have detectable histone peptide binding when tested with HEMP technology.

### Validation of candidate positives

Next, we investigated whether the novel interactions discovered in the screen could also be detected in conventional modified-histone binding assays. First, in biotinylated histone peptide pull-down assays, MPP8_CD_, TDRD7_TD_, and JMJ2C_TD_, all reproduced the binding activity observed in the screen (Fig. 4c). Second, GST-pull-down assays of purified bulk histones, GST-MPP8_CD_, GST-TDRD7_TD_, and GST-JMJ2C_TD_ proteins all pellet full-length histone H3, but GST does not (Fig. 4e). Moreover, we detected the cognate modification bound by each respective domain in the pellet – for example, JMJ2C_TD_ preferentially purified H3K4me3 versus H3K9me3 and TDRD7_TD_ preferentially pellets H3K9me3 (Fig. 4e). Finally, we found that MPP8_CD_ associates with nucleosomes purified from HeLa cells, preferentially interacting with nucleosomes enriched for H3K9me3 but not H3K4me3 (Fig. 4f; for quantification see Fig. S1). We note that due to homology to the CDY family, Fischle et al. suggested that MPP8_CD_, might bind to the ARK(S/T) motifs present around the H3K9 and H3K27 methylation sites [23] and although we did not detect an interaction with H3K27me on arrays, this interaction is observed in other *in vitro* binding assays (Fig. S2). Taken together, our results argue that MPP8_CD_, TDRD7_TD_, and JMJ2C_TD_ represent three new domains with specific histone PTM-binding activity and that HEMP technology can be used to identify and easily validate novel chromatin effectors.

## Discussion

Previously we demonstrated the utility of a modified histone peptide microarray to characterize methyl-lysine effector functions for the PHD fingers present within the yeast proteome [13]. Here we describe a human epigenome peptide microarray platform as a high-throughput tool for discovery of the factors that sense chromatin modifications. We focused our screen on the Royal domain super-family, testing greater than fifty domains from the chromodomain, PWWP, and Tudor families, as well as the majority of BRK, MRG, and SWIRM domains for binding to over sixty distinct modified peptides. All the domains tested are present on chromatin-associated human proteins. In our screen we detected the ten known modified-histone binders present in the library (CHD1_CD_, CDY1_ CD_, CDYL1_ CD_, CDYL2_ CD_, HP1α_ CD_, HP1β_CD_, HP1γ_CD_, 53BP1_ TD_, PHF20_TD_ and JMJ2A_ TD_) and discovered three novel methyl-histones binding modules: MPP8_CD_, TDRD7_TD_, and JMJ2C_TD_ (Table 1). We performed various histone-binding assays to independently validate the new interactions identified in the screen (Fig. 4c,e,f). Based on sequence alignment, MPP8_CD_, TDRD7_TD_, and JMJ2C_TD_ all appear to harbor a hydrophobic cage, the conserved molecular strategy for recognizing methyl-lysine (Fig. S3). Interestingly, TDRD7_TD_ is the first tudor domain described to date that preferentially binds to H3K9me2/3 versus the numerous other methyl-lysine sites on the arrays. Examination of the sequence reveals that the TDRD7_TD_ contains conserved sequence with other tudor domains at the residues that comprise the hydrophobic cage (Fig. S3a; highlighted with a green circle), but lacks the residue critical for H3K4me site specificity found in both JMJ2C and JMJ2A (Fig. S3a; highlighted with an orange circle). JMJ2C, which was identified here as an H3K4me-binder, functions as a histone lysine demethylase that removes one or more methyl moieties from H3K9me3 and H3K36me3 [27], [29], and its ability to bind to H3K4me might be important for regulating the dynamics of these other histone marks. The function of TDRD7 and the role of H3K9me-binding are not yet known and require future investigation.

Besides the positive interactions detected in the screen, we can also draw conclusions based on the modules that do not interact with any of the peptides present on the array. In this regard, there are several explanations as to why a domain may fail to give detectable signal on the HEMP array – the simplest explanation being that the ligand for the domain is not present on the slide (e.g. SMN_TD_). Additionally, it is possible that ligand-recognition by a candidate domain might only occur in the context of a nucleosome or require multiple modifications present within the ligand. Since HEMP is a versatile platform, meaning the peptide composition can be altered as new chromatin PTMs are discovered, peptides corresponding to new modifications can be easily synthesized and incorporated. This technique is also well-suited to study the combinatorial nature of chromatin modifications, since peptides can be produced with more than one modification [30]. While the peptide library generated here contains human histone sequences (Table S1) there are occasional differences in the primary amino acid structure of histones between organisms, so this technology can be adapted to study modification-dependent interactions in other organisms. Moreover, lysine methyl-transferases are evolutionarily conserved with more than 50 candidates in the human proteome – and it is probable that lysine methylation of non-histone proteins will emerge as a common mechanism for regulating signaling networks. For example, multiple lysine PTM sites contribute to various activities of p53 [31]–[33], and it is possible that novel binding partners for PTMs of p53 and other proteins in diverse organisms can be identified using array methods as described here.

Finally, in addition to the Royal family, there are several other domain families in which a subset of members is reported to have chromatin-effector functions. For example, the interaction between several PHD fingers and histones is regulated by lysine methylation [8]–[11], [15], [34], [35]. The ankryin repeats of G9A and GLP have also been shown to bind H3K9me [36]. As there are 150–300 PHD fingers and >300 ankryin repeats in the human proteome, HEMP technology can provide a rapid and reliable method for discerning potential chromatin effector functions for these modules. Moreover, the relative ease of diversifying the HEMP arrays to include additional modified peptides, dual-modified peptides, and non-histone modified peptides (e.g. methyl-lysine p53), will allow for the testing of numerous protein libraries, thus paving the way to discovery of domains with as yet to be defined activity. In summary, we have established array platforms for proteomic-scale discovery of the proteins that sense and transduce chromatin states into diverse biological readouts.

## Materials and Methods

### Human epigenetic peptide arrays

Biotinylated histone peptides were synthesized as described previously [10]. Peptides were printed in six replicates onto streptavidin-coated slides (ArrayIt) using the VersArray Compact Microarrayer (BioRad). All printed slides were air-dried overnight prior to use. Directly before use, unbound streptavidin sites were blocked with free biotin (Sigma; 1 mg/mL). Slides were incubated at 4°C overnight with GST-fusion proteins diluted in peptide binding buffer (50 mM Tris-HCl 7.5, 150 mM NaCl, 0.1% NP-40, 20% fetal bovine serum). Next, slides were washed 6 times with peptide binding buffer and probed with anti-GST antibody (Millipore) diluted in PBS containing 0.1% Tween-20 (PBST) and 20% FBS at room temperature for 1 hour. Slides were washed with PBST six times, then incubated 30 minutes with Alexa Fluor 647 chicken anti-rabbit IgG (Invitrogen) diluted in (PBST with 20% FBS). Lastly, slides were washed with PBST 6 times, briefly rinsed with PBS and air-dried. A GenePix 4000 scanner (Molecular Devices) was used to scan the arrays, and data images were analyzed by GenePix Pro Version 56.0 1 software.

### Heatmap analysis

To generate a heatmap representation of the data, the SNR (signal to noise ratio) column was first taken from the GPR (GenePix Report) files generated upon image analysis. GenePix 6.1 calculates SNR as the ratio of the mean net signal intensity (mean background pixel intensity subtracted from the mean foreground pixel intensity) over the standard deviation of the background pixel intensity. Because each antibody performs differently with respect to binding specificity on the array, the SNR for each array was normalized to a range of 0–100. These numbers were then converted to a heatmap with Java TreeView 1.13, with the peptides listed by row and the columns listed by antibody.

### Pull-down assays

Biotinylated histone pull-down assays were performed as performed previously [10]. Briefly, 1 ug of biotinylated peptides were incubated with 1 ug of GST-domain in peptide binding buffer (50 mM Tris-HCL, pH 7.5, 150 mM NaCl, 0.1% NonidetP-40) overnight at 4°C. After 1 h incubation with streptavidin beads (Amersham), complexes were washed 3 times with binding buffer, and the bound proteins were subjected to western analysis. Calf thymus (CT) histone pull-down and assays were performed as in [10]. Briefly, 10 ug of GST-domain was incubated with 50 µg CT histones (Worthington) in binding buffer (50 mM Tris-HCL, pH 7.5, 1 M NaCl, 1% NonidetP-40). After 1 h incubation with glutathione beads (Amersham), complexes were washed 3 times with binding buffer, and the bound proteins were subjected to western analysis. HeLa nucleosome pull-down assays were performed as reported in [15]. Briefly, 10 ug of GST-domain was incubated with 10 ug of purified HeLa nucleosomes in binding buffer (50 mM Tris pH 7.5, 150 mM NaCl, 0.1% NP-40, 10% glycerol). Incubation with glutathione beads and wash steps were the same as for CT histone pull-downs.

### Construction of chromatin domain expression library

Sequences were amplified from human cDNA and inserted into pDONR221. The sequence validated clones where subsequently subcloned into pDEST15 (Invitrogen). Domain boundaries were chosen based on SMART database entries [37] (see Table S4).

## Supporting Information

Figure S1 Quantitation of histone marks in HeLa nucleosomes pelleted by MPP8_CD_. ImageJ software was used for quantitative densitometric analysis of the gel band intensities shown in Figure 4f. For each antibody the pellet/input was calculated and plotted as a percentage.
(0.07 MB TIF)

Figure S2 *In vitro* association of MPP8_CD_ with H3K27me. (a) Histone peptide pull-downs indicate weak association with the chromodomain of MPP8 (MPP8_CD_) and H3K27me2/3. (b) Calf thymus histone (CTH) pull-down assay (top) and HeLa nucleosome pull-down assay (bottom) probed with H3K27me3. MPP8_CD_ precipitates H3K27me3 from HeLa nucleosomes but not CTH. Although we did not detect an interaction with H3K27me on the array, we performed additional *in vitro* binding assays as a result of a recent study [23] in which Fischle et al. suggest that CDs like the one present in MPP8_CD_ might bind ARK(S/T) motifs present at both the H3K9 and H3K27 methylation sites. We note that the binding of MPP8_CD_ to H3K27me is weaker that H3K9me when compared side-by-side in peptide pull-down assays.
(0.28 MB TIF)

Figure S3 Putative hydrophobic cage of MPP8, TDRD7, and JMJ2C. (a) Alignment of tudor domains that bind methyl-lysine: JMJ2A, JMJ2C, 53BP1, and TDRD7. An orange circle highlights Asp945 of JMJ2A_TD_. #appears at residues that when mutated diminish or ablate the H4K20me3-53BP1 tudor interaction [25]. * marks residues that when mutated diminish or ablate the interaction between the double tudor domain of JMJ2A with H3K4me3 [27]. (b) Alignment of chromodmains that bind H3K9me/27me: HP1, CDY, and MPP8. #indicates residues that when mutated diminish or ablate the interaction between the chromodomain of HP1 with H3K9me [19]. (a) and (b) Residues shaded in yellow are highly conserved in the region selected. A green circle marks residues that compose the hydrophobic cage of (a) JMJ2A [27] or (b) HP1 [19]. Residues shaded in blue are identical in the selected region. All sequences are those found in the human protein.
(0.99 MB TIF)

Table S1 HEMP Biotinylated Peptide library. Chemical modifications are indicated in parentheses after the modified residue. The location of the biotin is indicated by (bio). ac  =  acetyl-, me  =  methyl-, ph  =  phospho-
(0.07 MB DOC)

Table S2 Antibodies used to probe HEMP arrays.
(0.03 MB DOC)

Table S3 Comparison of histone marks detected on slide platform to dissociation constants determined in independent reports. CD  =  chromodomain; PHD  =  plant homeodomain; TD  =  tudor domain.
(0.08 MB DOC)

Table S4 Expression library of domains tested in this study.
(0.08 MB DOC)
