# Supplementary figures and images for: Epigenome Microarray Platform for Proteome-Wide Dissection of Chromatin-Signaling Networks
Source: PLoS One. 2009 Aug 26;4(8):e6789. doi: 10.1371/journal.pone.0006789 (PMC2777412; doi:10.1371/journal.pone.0006789)

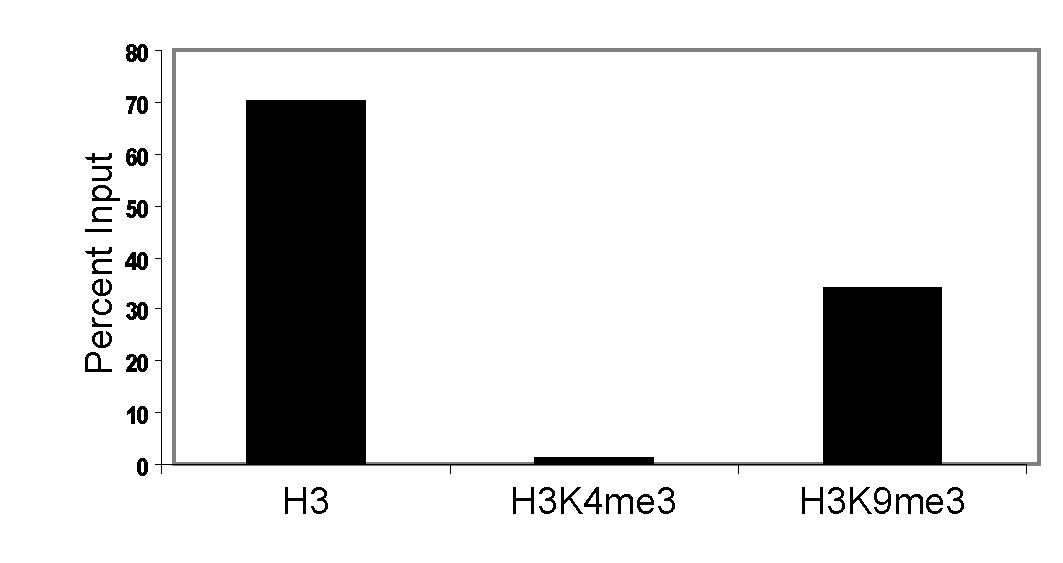

Supplement: Figure S1 — Quantitation of histone marks in HeLa nucleosomes pelleted by MPP8CD. ImageJ software was used for quantitative densitometric analysis of the gel band intensities shown in Figure 4f. For each antibody the pellet/input was calculated and plotted as a percentage. (0.07 MB TIF) [file pone.0006789.s001.tif]

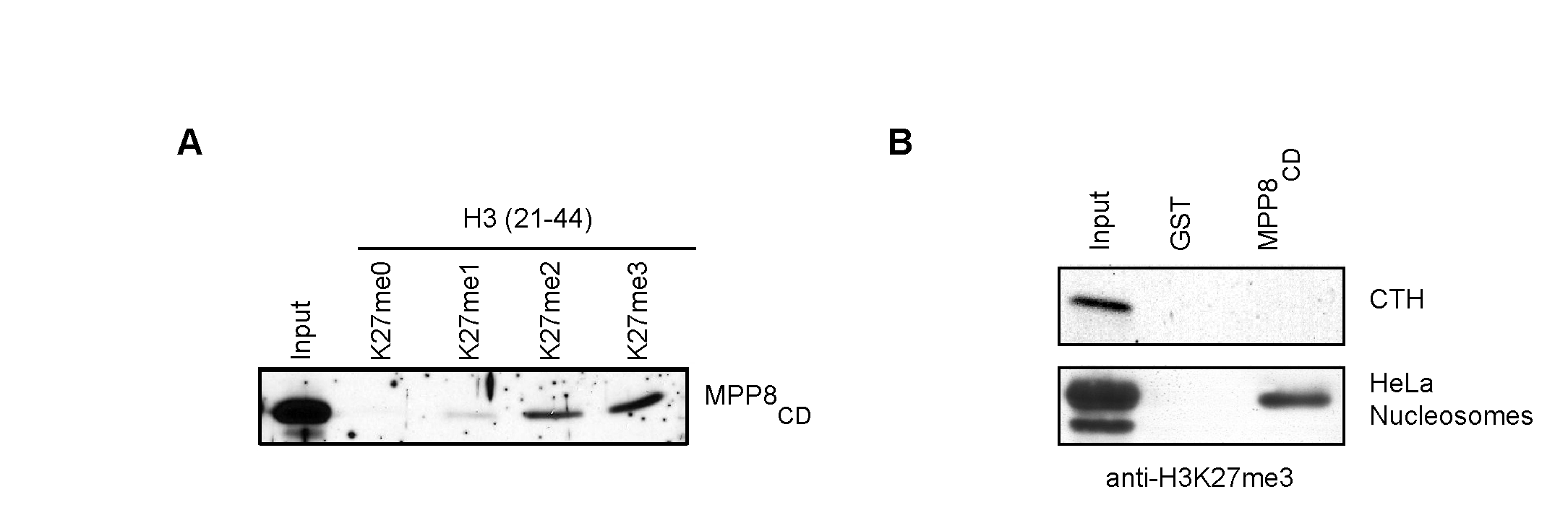

Supplement: Figure S2 — In vitro association of MPP8CD with H3K27me. (a) Histone peptide pull-downs indicate weak association with the chromodomain of MPP8 (MPP8CD) and H3K27me2/3. (b) Calf thymus histone (CTH) pull-down assay (top) and HeLa nucleosome pull-down assay (bottom) probed with H3K27me3. MPP8CD precipitates H3K27me3 from HeLa nucleosomes but not CTH. Although we did not detect an interaction with H3K27me on the array, we performed additional in vitro binding assays as a result of a recent study [23] in which Fischle et al. suggest that CDs like the one present in MPP8CD might bind ARK(S/T) motifs present at both the H3K9 and H3K27 methylation sites. We note that the binding of MPP8CD to H3K27me is weaker that H3K9me when compared side-by-side in peptide pull-down assays. (0.28 MB TIF) [file pone.0006789.s002.tif]

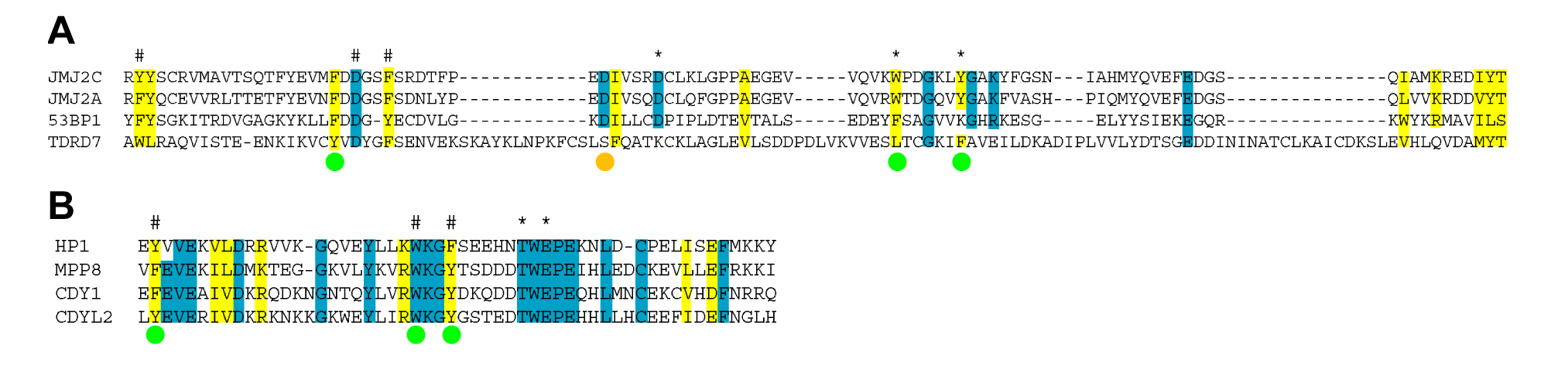

Supplement: Figure S3 — Putative hydrophobic cage of MPP8, TDRD7, and JMJ2C. (a) Alignment of tudor domains that bind methyl-lysine: JMJ2A, JMJ2C, 53BP1, and TDRD7. An orange circle highlights Asp945 of JMJ2ATD. #appears at residues that when mutated diminish or ablate the H4K20me3-53BP1 tudor interaction [25]. * marks residues that when mutated diminish or ablate the interaction between the double tudor domain of JMJ2A with H3K4me3 [27]. (b) Alignment of chromodmains that bind H3K9me/27me: HP1, CDY, and MPP8. #indicates residues that when mutated diminish or ablate the interaction between the chromodomain of HP1 with H3K9me [19]. (a) and (b) Residues shaded in yellow are highly conserved in the region selected. A green circle marks residues that compose the hydrophobic cage of (a) JMJ2A [27] or (b) HP1 [19]. Residues shaded in blue are identical in the selected region. All sequences are those found in the human protein. (0.99 MB TIF) [file pone.0006789.s003.tif]
