# Supplementary material for: Epigenome Microarray Platform for Proteome-Wide Dissection of Chromatin-Signaling Networks
Source: PLoS One. 2009 Aug 26;4(8):e6789. doi: 10.1371/journal.pone.0006789 (PMC2777412; doi:10.1371/journal.pone.0006789)
Supplement: Table S1 — HEMP Biotinylated Peptide library. Chemical modifications are indicated in parentheses after the modified residue. The location of the biotin is indicated by (bio). ac = acetyl-, me = methyl-, ph = phospho- (0.07 MB DOC) [file pone.0006789.s004.doc]

| **Peptide** | **Sequence** | **Modification** |
| --- | --- | --- |
| H2A (1-22) | sgrgkqggkarakaksrssragk(bio) |  |
| H2AX (121-142) | (bio)satvgpkapsggkkatqasqey |  |
| H2B (1-22) | pepaksapapkkgskkavtkaqk(bio) |  |
| H3 (1-21) | artkqtarkstggkaprkqlak(bio) |  |
| H3 (21-44) | atkaarksapstggvkkphryrpgk(bio) |  |
| H3 (44-64) | gtvalreirryqkstellirk(bio) |  |
| H3 (67-89) | tellirklpfqrlvreiaqdfktdlrfqsaaik(bio) |  |
| H4 (1-23) | (bio)sgrgkggkglgkggakrhrkvlr |  |
| H2AK5ac | sgrgk(ac)qggkarakaksrssragk(bio) | acetyl-lys |
| H2AK13ac | sgrgkqggkarak(ac)aksrssragk(bio) | acetyl-lys |
| H2BK5ac | pepak(ac)sapapkkgskkavtkaqk(bio) | acetyl-lys |
| H2BK12ac | pepaksapapkk(ac)gskkavtkaqk(bio) | acetyl-lys |
| H2BK15ac | pepaksapapkkgsk(ac)kavtkaqk(bio) | acetyl-lys |
| H2BK20ac | pepaksapapkkgskkavtk(ac)aqk(bio) | acetyl-lys |
| H3K9ac | Artkqtark(ac)stggkaprkqlak(bio) | acetyl-lys |
| H3K14ac | artkqtarkstggk(ac)aprkqlak(bio) | acetyl-lys |
| H3K18ac | artkqtarkstggkaprk(ac)qlak(bio) | acetyl-lys |
| H3K27ac | atkaarksapstggvkkphryrpgk(bio) | acetyl-lys |
| H3K56ac | gtvalreirryqk(ac)stellirk(bio) | acetyl-lys |
| H4K5ac | (bio)sgrgk(ac)ggkglgkggakrhrkvlr | acetyl-lys |
| H4K8ac | (bio)sgrgkggk(ac)glgkggakrhrkvlr | acetyl-lys |
| H4K12ac | (bio)sgrgkggkglgk(ac)ggakrhrkvlr | acetyl-lys |
| H4K16ac | (bio)sgrgkggkglgkggak(ac)rhrkvlr | acetyl-lys |
| H3R2me | ar(me1)tkqtarkstggkaprkqlak(bio) | methyl-arg |
| H3R2me2a | ar(me2a)tkqtarkstggkaprkqlak(bio) | methyl-arg |
| H3R2me2s | ar(me2a)tkqtarkstggkaprkqlak(bio) | methyl-arg |
| H3R17me | artkqtarkstggkapr(me1)kqlak(bio) | methyl-arg |
| H3R17me2a | artkqtarkstggkapr(me2a)kqlak(bio) | methyl-arg |
| H3R17me2s | artkqtarkstggkapr(me2s)kqlak(bio) | methyl-arg |
| H2BK5me1 | pepak(me1)sapapkkgskkavtkaqk(bio) | methyl-lys |
| H2BK5me2 | pepak(me2)sapapkkgskkavtkaqk(bio) | methyl-lys |
| H2BK5me3 | pepak(me3)sapapkkgskkavtkaqk(bio) | methyl-lys |
| H3K4me1 | artk(me1)qtarkstggkaprkqlak(bio) | methyl-lys |
| H3K4me2 | artk(me2)qtarkstggkaprkqlak(bio) | methyl-lys |
| H3K4me3 | artk(me3)qtarkstggkaprkqlak(bio) | methyl-lys |
| H3K9me1 | Artkqtark(me1)stggkaprkqlak(bio) | methyl-lys |
| H3K9me2 | Artkqtark(me2)stggkaprkqlak(bio) | methyl-lys |
| H3K9me3 | Artkqtark(me3)stggkaprkqlak(bio) | methyl-lys |
| H3K18me1 | artkqtarkstggkaprk(me1)qlak(bio) | methyl-lys |
| H3K18me2 | artkqtarkstggkaprk(me2)qlak(bio) | methyl-lys |
| H3K18me3 | artkqtarkstggkaprk(me3)qlak(bio) | methyl-lys |
| H3K23me1 | atk(me1)aarksapstggvkkphryrpgk(bio) | methyl-lys |
| H3K23me2 | atk(me2)aarksapstggvkkphryrpgk(bio) | methyl-lys |
| H3K23me3 | atk(me3)aarksapstggvkkphryrpgk(bio) | methyl-lys |
| H3K36me1 | atkaarksapstggvk(me1)kphryrpgk(bio) | methyl-lys |
| H3K36me2 | atkaarksapstggvk(me2)kphryrpgk(bio) | methyl-lys |
| H3K36me3 | atkaarksapstggvk(me3)kphryrpgk(bio) | methyl-lys |
| H3K79me1 | tellirklpfqrlvreiaqdfk(me1)tdlrfqsaaik(bio) | methyl-lys |
| H3K79me2 | tellirklpfqrlvreiaqdfk(me2)tdlrfqsaaik(bio) | methyl-lys |
| H3K79me3 | tellirklpfqrlvreiaqdfk(me3)tdlrfqsaaik(bio) | methyl-lys |
| H4K20me1 | sgrgkggkglgkggakrhrk(me1)vlrk(bio) | methyl-lys |
| H4K20me2 | sgrgkggkglgkggakrhrk(me2)vlrk(bio) | methyl-lys |
| H4K20me3 | sgrgkggkglgkggakrhrk(me3)vlrk(bio) | methyl-lys |
| H2AXS139ph | (bio)satvgpkapsggkkatqas(ph)qey | phospho-ser |
| H3T3ph | art(ph)kqtarkstggkaprkqlak(bio) | phospho-thr |
| H3S10ph | artkqtarks(ph)tggkaprkqlak(bio) | phospho-ser |
