# Supplementary material for: Epigenome Microarray Platform for Proteome-Wide Dissection of Chromatin-Signaling Networks
Source: PLoS One. 2009 Aug 26;4(8):e6789. doi: 10.1371/journal.pone.0006789 (PMC2777412; doi:10.1371/journal.pone.0006789)
Supplement: Table S2 — Antibodies used to probe HEMP arrays. (0.03 MB DOC) [file pone.0006789.s005.doc]

| **Epitope** | **Manufacturer** | **Catalog Number** | **Lot Number** |
| --- | --- | --- | --- |
| Acetyl-Lysine | Upstate | 06-933 | 26409 |
| H3K9ac | Sigma | H9286 | 072K4824 |
| H3K18ac | Abcam | Ab1191 | 122108 |
| H3R2me2 | Abcam | Ab8046 | 11666 |
| H3K4me3 | Upstate | 07-473 | 27343 |
| H3K9me1 | Abcam | Ab9045 | 291918 |
| H3K36me3 | Abcam | Ab9050 | 446805 |
| H3K79me3 | Abcam | Ab2621 | 15113 |
| H4K20me3 | Abcam | ab9053 | 232666 |
| H4K20me3 | Upstate | 07-463 | 31392 |
| gammaH2AX | Abcam | Ab18311 | 305123 |
| gammaH2AX | Upstate | 05-636 | JBC1367868 |
