# Supplementary material for: Epigenome Microarray Platform for Proteome-Wide Dissection of Chromatin-Signaling Networks
Source: PLoS One. 2009 Aug 26;4(8):e6789. doi: 10.1371/journal.pone.0006789 (PMC2777412; doi:10.1371/journal.pone.0006789)
Supplement: Table S3 — Comparison of histone marks detected on slide platform to dissociation constants determined in independent reports. CD = chromodomain; PHD = plant homeodomain; TD = tudor domain. (0.08 MB DOC) [file pone.0006789.s006.doc]

| **Protein** | **Domain** | **Histone mark** | **Dissociation constant (microMolar)** | **Reference** | **Detected on Array** |
| --- | --- | --- | --- | --- | --- |
| CDY | CD | H3K9me1 | 3.4 | [23] | + |
|  |  | H3K9me2 | 0.7 | [23] | + |
|  |  | H3K9me3 | 0.5 | [23] | + |
|  |  | H3K27me1 | 300 | [23] | + |
|  |  | H3K27me2 | 119 | [23] | + |
|  |  | H3K27me3 | 76 | [23] | + |
| CDYL2 | CD | H3K9me1 | 67 | [23] | + |
|  |  | H3K9me2 | 8.9 | [23] | + |
|  |  | H3K9me3 | 3.9 | [23] | + |
|  |  | H3K27me1 | 113 | [23] | - |
|  |  | H3K27me2 | 18.4 | [23] | + |
|  |  | H3K27me3 | 12.4 | [23] | + |
| CHD1 | CD | H3K4me1 | 15.1 | [36] | + |
|  |  | H3K4me2 | 5.0 | [36] | + |
|  |  | H3K4me3 | 6.1 | [36] | + |
| dsHP1 α | CD | H3K9me1 | 46 | [20] | + |
|  |  | H3K9me2 | 7 | [20] | + |
|  |  | H3K9me3 | 4 | [20] | + |
| AIRE | PHD | H3 (1-21) | 10.6 | [14] | + |
| ING1 | PHD | H3K4me1 | 419 | [35] | + |
|  |  | H3K4me2 | 17.3 | [35] | + |
|  |  | H3K4me3 | 3.3 | [35] | + |
| ING2 | PHD | H3K4me1 | 208 | [34] | + |
|  |  | H3K4me2 | 15 | [34] | + |
|  |  | H3K4me3 | 1.5 | [34] | + |
| ING3 | PHD | H3K4me3 | 6.9 | [34] | + |
| ING4 | PHD | H3K4me3 | 7.9 | [34] | + |
| ING5 | PHD | H3K4me3 | 2.4 | [34] | + |
| RAG2 | PHD | H3K4me2 | 173.2 | [30] | + |
|  |  | H3K4me3 | 33.8 | [30] | + |
| 53BP1 | TD | H3K79me2 | 2000 | [25] | - |
|  |  | H4K20me1 | 52.9 | [25] | + |
|  |  | H4K20me2 | 19.7 | [25] | + |
|  |  | H4K20me3 | 1000 | [25] | - |
| JMJ2A | TD | H3K4me3 | 10.4 | [27] | + |
