# Supplementary material for: Epigenome Microarray Platform for Proteome-Wide Dissection of Chromatin-Signaling Networks
Source: PLoS One. 2009 Aug 26;4(8):e6789. doi: 10.1371/journal.pone.0006789 (PMC2777412; doi:10.1371/journal.pone.0006789)
Supplement: Table S4 — Expression library of domains tested in this study. (0.08 MB DOC) [file pone.0006789.s007.doc]

| **Protein** | **Domain** | **Accession #** | **Region** |
| --- | --- | --- | --- |
| CHD6 | BRK | NM_032221 | 2392-2452 |
| CHD7 | BRK | NM_017780 | 1792-1934 |
| CHD8 | BRK | NM_020920 | 2021-2150 |
| CHD9 | BRK | NM_025134 | 2472-2610 |
| SMCA2 | BRK | NM_003070 | 576-640 |
| SMCA4 | BRK | NM_003072 | 602-666 |
| ARID4A | CD | NM_002892 | 578-650 |
| CBX1 | CD | NM_006807 | 1-185 |
| CBX2 | CD | NM_005189 | 1-80 |
| CBX3 | CD | NM_016587 | 1-184 |
| CBX4 | CD | NM_003655 | 1-79 |
| CBX5 | CD | NM_012117 | 1-189 |
| CBX6 | CD | NM_014292 | 1-79 |
| CBX7 | CD | NM_175709 | 1-79 |
| CBX8 | CD | NM_020649 | 1-79 |
| CDY1 | CD | AAD22735 | 1-71 |
| CDYL1 | CD | NM_170751 | 1-77 |
| CDYL2 | CD | NM_152342 | 1-77 |
| CHD1 | CD | NM_001270 | 260-462 |
| CHD2 | CD | CR627422 | 250-352 |
| CHD3 | CD | NM_005852 | 496-693 |
| CHD4 | CD | NM_001273 | 489-687 |
| CHD7 | CD | NM_017780 | 27-115 |
| CHD8 | CD | NM_020920 | 351-521 |
| MPP8 | CD | NM_017520 | 46-121 |
| MSL3L1 | CD | NM_078629 | 1-100 |
| MRG15 | CD | AAD29872 | 1-80 |
| SMRC1 | CD | NM_003074 | 205-271 |
| SMRC2 | CD | NM_003075 | 176-245 |
| SUV91 | CD | NM_003173 | 32-104 |
| TIP60 | CD | NM_182710 | 1-86 |
| MO4L1 | MRG | NM_206839 | 1-363 |
| MO4L2 | MRG | NM_012286 | 1-289 |
| BRD1 | PWWP | NM_014577 | 917-1022 |
| HDGF | PWWP | NM_004494 | 1-91 |
| HDGR2 | PWWP | AB208892 | 1-90 |
| HDGR3 | PWWP | NM_016073 | 1-90 |
| MBD5 | PWWP | NM_018328 | 1372-1455 |
| NP60 | PWWP | BC032855 | 1-88 |
| NSD1 | PWWP | NM_022455 | 310-414 |
| PSIP1 | PWWP | NM_033222 | 1-86 |
| ZMYND11 | PWWP | AY732489 | 147-233 |
| ADA2 | SWIRM | NM_001488 | 346-444 |
| AOF1 | SWIRM | AK091428 | 62-180 |
| MYSM1 | SWIRM | AB067502 | 362-480 |
| SMRC1 | SWIRM | NM_003074 | 439-556 |
| SMRC2 | SWIRM | NM_003075 | 414-531 |
| 53BP1 | TD | NP_005648 | 1484–1603 |
| AKAP1 | TD | NM_003488 | 746-827 |
| ARI4A | TD | NM_002892 | 48-124 |
| ARI4B | TD | BC104632 | 48-124 |
| JMJ2A | TD | NP_055478 | 856-966 |
| JMJ2B | TD | NM_015015 | 907-1041 |
| JMJ2C | TD | NM_015061 | 867-1001 |
| LBR | TD | NM_002296 | 1-73 |
| MTF2 | TD | NM_007358 | 1-111 |
| PHF1 | TD | NM_002636 | 1-96 |
| PHF19 | TD | BX640713 | 1-105 |
| PHF20 | TD | NP_057520 | 85-129 |
| PHF20L1 | TD | NM_198513 | 75-151 |
| SETB1 | TD | NM_012432 | 246-411 |
| SMN | TD | NM_017411 | 80-161 |
| SND1 | TD | NM_014390 | 718-797 |
| STK31 | TD | NM_031414 | 67-147 |
| TDRD1 | TD | NM_198795 | 128-655 |
| TDRD3 | TD | Q5VUZ2 | 544-625 |
| TDRD7 | TD | NM_014290 | 503-770 |
